# The Attitude of Physicians Towards Female Genital Cosmetic Surgery

**DOI:** 10.7759/cureus.27902

**Published:** 2022-08-11

**Authors:** Dana Sawan, Malak Al-Marghoub, Ghaliah H Abduljabar, Mohammed Al-Marghoub, Faisal Kashgari, Nashwa Aldardeir, Hassan S Abduljabbar

**Affiliations:** 1 Obstetrics and Gynecology, King Abdulaziz University Faculty of Medicine, Jeddah, SAU; 2 Obstetrics and Gynecology, King Abdulaziz University Hospital, Jeddah, SAU; 3 Medicine, King Abdulaziz University Faculty of Medicine, Jeddah, SAU; 4 Plastic Surgery, King Abdullah University Hospital, Riyadh, SAU; 5 Obstetrics and Gynecology, University of Jeddah, Jeddah, SAU

**Keywords:** female genital cosmetic surgery, rejuvenation, labiaplasty, vaginoplasty, physicians, attitudes

## Abstract

Introduction

Female genital cosmetic surgery (FGCS) is a trending topic in the field of gynecology practice. It is defined as any genital procedure that is not medically indicated but is designed to improve the appearance of the genitals. With the increasing demand for FGCS, little is known about the attitudes of physicians, especially gynecologists, toward FGCS. The study objective is to assess physicians' attitudes towards female genital cosmetic surgeries in Jeddah, Saudi Arabia.

Methods

A cross-sectional survey was done among physicians in Jeddah, Saudi Arabia. Four-sectioned questionnaires were distributed to physicians. All board-certified consultants and registrars of obstetrics and gynecology, plastic, and reconstructive surgery in private/public hospitals were included in the study. Data management was done using the SPSS program version 19 (IBM Corp., Armonk, NY, USA).

Results

Out of 165 physicians, 40 were practicing physicians (female genital cosmetic surgery) (24.2%) while 125 were non-practicing physicians (75.8%). The age of practicing and non-practicing respondents ranges from 26 to 60 with a mean and standard deviation (42.6±8.6) and 24 to 60 (40.1±8.9) years old. Our study showed that there were females under 18 years old requesting FGCS from all physicians. Results of attitude towards FGCS showed that the majority of non-practicing physicians were more comfortable in providing advice about FGCS (N=53) and very confident about female anatomy (N=65). Non-practicing physicians also knew a lot more about the long and short-term risks of FGCS and discussed them all the time with their patients. In terms of giving advice on labiaplasty, clitoral hood reduction, perineoplasty, hymenoplasty, and G-spot augmentation procedures, practicing physicians were more confident compared to non-practicing physicians. Statistical analysis showed a significant difference between practicing and non-practicing physicians in terms of gender (p=0.001), career level (p=0.005) and type of work (p=0.006), giving advice on labiaplasty (p=0.001), clitoral hood reduction (p=0.001), perineoplasty (p=0.016) and G-spot augmentation (p=0.001), the number of patients per month, patients seeking advice about FGCS, knowledge about short-term and long-term risks and possible risks of FGCS, vaginal examination, offering referral/counseling and confidence in assessing female anatomy (all, p=0.001).

Conclusion

Physicians in Jeddah, Saudi Arabia showed an overall positive attitude towards FGCS. The study suggests that with enough training and knowledge about FGCS, physicians could exhibit a more positive attitude towards FGCS.

## Introduction

Female genital cosmetic surgery (FGCS) nowadays is a trending topic in the field of gynecology practice. It is defined as any genital procedure that is not medically indicated but is designed to improve the appearance of the genitals. It is claimed that, after FGCS, sexual function may be improved [1]. The study objective is to assess physicians' attitudes towards female genital cosmetic surgeries in Jeddah, Saudi Arabia.

It was described by Hodgkinson and Hait in 1984 on cosmetic labiaplasty procedures that FGCS includes labia minora reductions, vaginal tightening (“rejuvenation”), labia majora “augmentations”, pubic liposuction (mons pubis, labia majora), clitoral hood reductions, hymen “reconstruction”, perineum “rejuvenation”, and “G-spot amplification”, but not limited to that [2,3].

According to the American Society for Aesthetic Plastic Surgery, labiaplasty surgery (excluding vaginal rejuvenation) has increased by 23.2% from 2015 to 2016. Around half of the surgeries, 51.1%, were done on middle-aged patients ranging from 19 to 34 years old [3].

This increase in awareness for cosmetic surgery is most likely caused by media, in a research done by Crockett et al. about the influence of plastic surgery they found that four out of five patients revealed that TV shows influenced them to seek a cosmetic procedure, not to mention the high expectations after this kind of surgeries [4-7].

Trending of these surgeries will stress the physician to learn more about it and definitely the personal opinion of each physician will affect his own concept of a healthy vagina picture and his readiness in performing it [6].

In fact, this topic cannot be ignored by a general physician and especially gynecologist doctors, whether they agree with aesthetic gynecology or not, they will be consistently counseled by women who are dissatisfied or concerned about the appearance of their genitalia. 

In order to organize the ethical dilemma in such a matter, the Royal College of Obstetricians and Gynecologists (RCOG), as well as the American College of Obstetricians and Gynecologists (ACOG), have released a paper about ethical considerations in relation to labial surgery. Although there are no controlled studies done to measure the effectiveness and long-term complications of such procedures, the first and most important step is to reassure and demonstrate to women requesting FGCS about the normal variations in female genitalia. Patients should also be screened and counseled about psychological body image problems, such as body image distress, and the treatment options for these problems. Sometimes a referral to a mental health professional or a psychiatrist is needed to fulfill a dull body image satisfaction [8,9].

There have been a number of researches done to assess the motivation behind women seeking FCGC. However, much uncertainty still exists about physician attitude and knowledge toward FGCS. In a study done in Australia to assess general physicians’ knowledge and attitude toward FGCS, they found that 75% of general physicians feel like their knowledge is inadequate and their primary sources of information are basically the conferences, other physicians and media, respectively [10].

In the light of such information, the study aimed to assess physicians’ attitudes towards female genital cosmetic surgeries in Saudi Arabia and to evaluate patient demand. The current study will contribute to our knowledge by addressing the following issues. First, to assess general gynecologists’ attitudes toward FCGS, and how comfortable they feel when being asked to counsel about it or perform it. Second, to assess general gynecologists’ knowledge about FGCS, and where they got their knowledge from.

## Materials and methods

Study design, settings, and participants

The study design, setting and time frame were a cross-sectional survey done in Jeddah, Saudi Arabia from May to June 2019. The survey was distributed among general gynecologists and doctors of other specialties. The inclusion criteria were physicians from private and public hospitals in Jeddah of board-certified obstetrics and gynecology, plastic and reconstructive surgery registrars, and consultants. The exclusion criteria were other specialties, medical students and hospital administrators. The emails were requested from the HR department of major hospitals in Jeddah, Saudi Arabia, and the questionnaires were sent to the participating doctors. Population size was around 250 physicians in the major hospitals, and when calculating the sample size it turned out to be 165. From the 165 physicians, only 40 were performing female genital cosmetic surgeries and they were labeled "practicing physicians". Participating physicians were those who accepted patients needing cosmetic gynecology, and performed cosmetic surgery. On the other hand were the non-practicing physicians, who do not perform female genital cosmetic surgery but are specialized in other fields of obstetrics and gynecology and accept patients seeking cosmetic gynecology for consultation only.

Data collection methods 

Data collection was carried out by a pre-designed questionnaire. The questionnaire included four sections. The first section was about socio-demographic data (gender, age, specialty, career level, type of work, degree), their background knowledge, and practice. The second part contained questions about FGCS, such as labiaplasty, clitoral hood reduction, perineoplasty, hymenoplasty, orgasm shot, vaginal rejuvenation, vulval liposuction, and G-spot augmentation. It addressed how confident the physicians feel when giving advice about the FGCS procedures, and how comfortable they feel when they give advice. The questionnaire also included how many patients they see every week, month, or year asking for FGCS, and the ages of women asking about it. The third section asked about the long- and short-term risks of FGCS, and if they talk about the possible risks with the patients.

Data analysis

Data analysis was done using IBM SPSS software version 19 (IBM Corp., Armonk, NY, USA). Results were presented as counts and percentages for categorical and nominal values while continuous variables were presented by mean and standard deviations. Statistical analysis was done to determine the difference between the two groups (practicing and non-practicing physicians). P-value <0.05 was considered significant.

Ethical approval

The questionnaire was distributed among physicians after the IRB approval from the research unit of biomedical ethics at King Abdulaziz University, under the IRB approval reference number 618-19. The approval was obtained on October 2, 2019.

## Results

Sociodemographic data

In the current study, attitudes towards FGCS among physicians in Saudi Arabia were gathered and assessed. Out of 165 physicians, 117 were female (70.9%) while 48 were male (29.1%) as shown in Table 1. The majority of the respondents specialized in obstetrics-gynecology (N=136, 82.4%). In regards to career level, most of the respondents were consultants (N=87, 52.7%), followed by registrars (specialists) (N=35, 21.2%), and junior residents (N=22, 13.3%). Moreover, professors (N=11, 6.7%) and senior residents (N=10,6.1%) were the least respondents. Furthermore, 50.9% of the physicians (N=84) worked in private institutions, and 49.1% (N=81) worked in non-private institutions. Only 24.2% of the physicians (N=40) were currently practicing, while the majority of the respondents 75.8% (N=125) were non-practicing FGCS obstetrics and gynecology physicians. 

**Table 1:** Demographic characteristics of 165 physicians in Saudi Arabia.

Characteristics	N	%
Gender		
Female	117	70.9
Male	48	29.1
Specialty		
Obstetrician-Gynecologist	136	82.4
Non-Obstetrician-Gynecologist	29	17.6
Level of education		
Consultant	87	52.7
Registrar (specialist)	35	21.2
Junior resident	22	13.3
Professor	11	6.7
Senior resident	10	6.1
Work		
Non - Private	81	49.1
Private	84	50.9
Practicing female genital cosmetic surgery (FGCS)		
Yes	40	24.2
No	125	75.8

Comparison among the participating physicians

As shown in Table 2, practicing physicians were in the range of 26 to 60 years, with a mean age of 42.6±8.6 years. For non-practicing physicians, age ranged from 24 to 60 years with a mean age of 40.1±8.9 years. Regarding their source of information about FGCS, the majority of the 40 practicing physicians got FGCS information from the media and online courses (N=16, 40%), and then from conferences. Moreover, most of the 125 non-practicing physicians were obstetrics and gynecology consultants, and they got their knowledge about FGCS mainly from the conferences they attended (N=77, 61.5%). 

**Table 2:** Comparison of demographic characteristics and source of information between practicing and non-practicing physicians.

Variables	Practicing	Non-practicing	Odd ratio	p value
	40 (24.2%)	125 (75.8%)		
Age (mean ± SD)	Range: (26-60) 42.6±8.6	Range: (24-60) 40.1±8.9		0.129
Gender				
Female	19	98	0.249 (0.117- 0.529)	0.001
Male	21	27		
Specialty				
Non-Obstetrician-Gynecologist	12	27	2.723 (1.166 6.356)	
Obstetrician	28	108		0.019
Level of education				
Consultant	17	70		
Junior resident	11	11		0.005
Professor	1	10		
Registrar	6	29		
Senior resident	5	5		
Work				
Non private	27	54	2.731 (1.289 5.783)	0.006
Private	13	71		
Information source				
Conference	12	77		0.001
Job	1	4		
Media and online courses	16	36		
Others	11	8		

A comparison between the demographic characteristics of practicing physicians and the non-practicing ones was done. The results revealed that there was a statistically significant difference in regards to gender (p=0.001), career level (p=0.005), and type of work (p=0.006) with odds ratios of 0.249, 2.731 for gender and career level respectively. A significant difference was also observed between practicing and non-practicing physicians in regards to their source of information. On the other hand, no significant difference in age or specialty was observed. 

Attitudes of physicians towards giving advice on different genital procedures is shown in Table 3. It was found that practicing physicians were more confident in giving advice about labiaplasty, clitoral hood reduction, perineoplasty, hymenoplasty, and G-spot augmentation compared to non-practicing physicians. In terms of counseling about vaginal rejuvenation, both practicing and non-practicing physicians were equally confident. On the other hand, all physicians were not confident in giving advice on the orgasm shot procedures. Statistical analysis showed that there was a significant difference between practicing and non-practicing physicians in terms of giving advice on labiaplasty (p=0.001), clitoral hood reduction (p=0.001), perineoplasty (p=0.016), and G-spot augmentation (p=0.001) procedures with odd ratios of 0.175, 0.014, 0.423, 0.300, respectively.

**Table 3:** Attitudes of physicians regarding giving advice on different procedures.

Variables	Practicing	Non-practicing	Odd ratio	p value
	40 (24.2% )	125 (75.8%)		
Labiaplasty				
Not confident	9	178	0.175 (0.077-0.399)	0.001
Confident	31	47		
Clitoral hood reduction				
Not confident	12	121	0.014 (0.004-0.047)	0.001
Confident	28	4		
Perineoplasty				
Not confident	4	701	0.423 (0.202 - 0.886)	0.016
Confident	26	55		
Hymenoplasty				
Not confident	9	651	0.835 (0.409 - 1.704)	0.377
Confident	21	60		
Orgasm shot (O-shot)				
Not confident	25	922	0.598 (0.281 - 1.270)	0.127
Confident	15	33		
Vaginal rejuvenation				
Not confident	6	521	0.936 (0.453 - 1.934)	0.504
Confident	24	73		
G-spot augmentation				
Not confident	13	771	0.3 (0.141 - 0.638)	0.001
Confident	27	47		

In terms of the number of patients requesting FGCS per month, practicing physicians had one to 20 patients while non-practicing obstetrics and gynecology physicians have 0 to 20 patients per month. Results also revealed that there were patients under 18 years old requesting FGCS. When giving advice about FGCS, most non-practicing obstetrics and gynecology physicians were comfortable (N=53) out of 125, while 27 out of 40 of the practicing physicians were comfortable. Regarding the risk of surgery, it was observed that there is no statistically significant difference regarding the short- and long-term risks.

**Table 4:** Attitudes of physicians towards female genital cosmetic surgery (FGCS) and risks of surgery

Variables	Practicing	Non-practicing	Odd ratio	p value
	40 (24.2%)	125 (75.8%)		
Number of patients per month	Range: (1-20) 9.9 ±3.79	Range: (0-20) 4.3±3.96		0.001
Number of patients less than 18 years				
No	33	93	1.622 (0.654-4.026)	0.204
Yes	7	32		
Age range of patients seen				
20-25	11	17		0.276
25-30	4	11		
30-35	10	46		
35-40	10	28		
40-45	3	8		
45-50	2	15		
Advice FGCS				
Comfortable	27	53	2.82 (1.33-5.98)	0.005
Not comfortable	13	72		
Do you know about short term risk?				
A lot	23	65	1.249 (0.609-2.562)	0.336
Not at all	17	60		
Do you know about long term risk?				
A lot	21	56	1.362 (0.667-2.780)	0.252
Not at all	19	69		

Table 5 showed that both practicing (N=23) and non-practicing physicians (N=93) performed vaginal examinations. The majority of the practicing (N=13) and non-practicing physicians (N=62) also offer referral/counseling. Regarding their confidence in assessing the female anatomy, most practicing physicians were confident (N=20), while the majority of the non-practicing obstetrics and gynecology respondents were very confident (N=65). In regards to how much teaching regarding female genital anatomy was received during medical training, the majority of practicing (N=18) and non-practicing physicians (N=71) received a lot. Further analysis showed that there was a significant difference between the attitudes of practicing and non-physicians in terms of vaginal examination, offering referral/counseling, and confidence in assessing female anatomy (all, p=0.001). 

**Table 5:** Physicians’ education and practice

Variables	Practicing	Non-practicing	p value
	40 (24.2%)	125 (75.8%)	
Do you do vaginal exam?			
No	13	15	0.001
Not applicable	0	3	
Sometimes	4	14	
Yes	23	93	
Do you offer referrals?			
No	12	31	0.001
Not applicable	9	4	
Sometimes	6	28	
Yes	13	62	
Do you feel confident about female anatomy?			
Confident	20	50	0.001
Not confident	12	10	
Very confident	8	65	
How much training			
A little	5	20	0.255
A lot	18	71	
None	4	5	
Some	13	29	

## Discussion

In this study, physicians in Saudi Arabia showed an overall positive attitude towards FGCS. The majority of the physicians who responded to the survey were females, specializing in obstetrics-gynecology and at the consultant level. Also, most of the respondents were obstetrics and gynecology physicians, who do not practice FGCS, working in private institutions. 

Obstetrics and gynecology physicians in this survey were classified into two types: practicing and non-practicing physicians. This survey was designed to observe the differences between these two groups. Demographic characteristics and source of FGCS information of all physicians were statistically compared. Results showed that only gender, career level, and type of work were statistically different between practicing and non-practicing physicians. In terms of source of information, most of the practicing physicians knew about FGCS from the media and online workshops mainly, conferences and other sources, while the majority of the non-practicing physicians were consultants and got their information from conferences. 

Findings showed that practicing physicians were more confident in giving advice on different genital procedures such as labiaplasty, clitoral hood reduction, perineoplasty, hymenoplasty, and G-spot augmentation. The results were similar to the American survey, in which the majority of the physicians accepted labiaplasty requests [10]. However, in a European study, physicians were more conservative in performing or offering such procedures [11]. In a Turkish study, physicians stated that there was a rare medical justification for performing hymenoplasty, G-spot augmentation, and clitoral hood reduction procedures [12]. This implies that culture and geography may affect physicians in performing or referring genital procedures. 

With regards to age range, it was observed that the majority of the females seen by practicing physicians were between 20 and 25 years, while it ranged from 30 to 35 years for non-practicing physicians. These results were similar to the American 2016 survey [3]. Demand for FGCS was also observed in teenagers worldwide. This was evident in the study in which females under 18 years old were requesting FGCS. According to different groups and organizations, FGCS should not be conducted in females younger than 18 years old [13,14]. Based on the Royal Australian College of General Practitioners (RACGP) guidelines, FGCS can be done in patients under 18 years old but they should be referred to a physician specializing in adolescent gynecology. 

Findings further revealed that physicians were overall confident in giving advice to women asking for FGCS. The majority of practicing physicians were more confident than non-practicing physicians. This positive attitude of practicing physicians may be attributed to their experience in the field and the knowledge from the conference they have attended. Also, both practicing and non-practicing physicians knew equally about the short-term and long-term risk of FGCS, which could be similar to other procedures' risks. They also discussed the possible risks of FGCS to their patients all the time. This kind of practice was in-line with RACGP recommendations in which physicians were required to discuss the FGCS procedures including risks and possible complications to their patients [13].

This study has some potential limitations. It is a cross-sectional study in which selection and socially desirable biases could be possible. Nevertheless, the sample size is large, thus it could give conclusive results and could be potentially used in future studies. Second, there was a noted difference between the number of female physicians and male physicians who responded which could possibly lead to response bias. Finally, the practicing responders were much fewer than the non-practicing. 

## Conclusions

Gynecologists in Jeddah, Saudi Arabia showed an overall positive attitude towards FGCS. A wide variety of female patients tend to ask their obstetrics and gynecology physicians about FGCS counseling. In our study the majority were obstetrics and gynecology consultants who were not practicing FGCS, however their knowledge enabled them to counsel their patients properly. This was done by informing them about long- and short-term consequences, vaginally examining them, and by referring them to the practicing physicians. Practicing and non-practicing physicians are an important source of information, and could play an important role in educating patients. The results showed an overall positive attitude towards FGCS. 

## Appendices

Attitude of physicians towards female genital cosmetic surgery

1. Demographic data:

Age _______ Gender _________ Speciality (Ob\plastic) Sub-speciality _______________ In which country did you complete your professional degree ____________ Years of practice _______

2. Knowledge and practice. (please circle)

- Have you seen patients who have asked you about any form of FGCS? Yes / No - Where have you acquired information about FGCS from? (tick all that apply) o Media o Conferences  

o Other health professionals o Internet websites o Other ___________________________

- How confident do you feel to give advice for each of the following procedures: • labiaplasty? Not confident/Reasonably confident/Very confident • clitoral hood reduction? Not confident/Reasonably confident/Very confident • perineoplasty? Not confident/Reasonably confident/Very confident • hymenoplasty? Not confident/Reasonably confident/Very confident • orgasm shot ( O-shot) ? Not confident/Reasonably confident/Very confident • vaginal rejuvenation ? Not confident/Reasonably confident/Very confident • vulval liposuction? Not confident/Reasonably confident/Very confident • G spot augmentation Not confident/Reasonably confident/Very confident

- How comfortable do you feel advising a woman who asks for FGCS? o Not comfortable o Comfortable o Very comfortable  

- How many patients do you see requesting FGCS? _____ per week _____per month ______per year

- Have you had any patients less than 18 years old asking you about FGCS? Yes / No - What is the age range of women asking you about FGCS? o Not applicable. I haven’t had any patients asking me about FGCS. Youngest: ________ years  

Oldest: __________ years

 
3. FGCS and risks of surgery - Do you know about the possible short term risks of FGCS? o Not at all oAlittle oAlot - Do you know about the possible long term risks of FGCS? o Not at all oAlittle oAlot - Do you talk about possible risks of FGCS for patients who request this? o Not applicable. I haven’t seen a patient asking for FGCS o Only if they ask me o Sometimes o All the time

- Have you been asked for (tick all that apply): o an examination? o your opinion ? o a referral?

- Do you examine these women’s genital area ? o Yes o No o Sometimes 

o Not applicable (I haven’t seen any patients asking about FGCS)

Attitude of physicians towards female genital cosmetic surgery

- Do you offer / refer for counselling? o Yes o No o Sometimes 

o Not applicable (I haven’t seen any patients asking about FGCS)

- Which specialist group have you referred women to for FGCS? (tick all that apply)   o Plastic Surgeon o Obstetrician Gynaecologist o Urologist 

o Psychologist / Psychiatrist o Colleague for second opinion o Women’s health GP o Not applicable (I haven’t seen any patients asking about FGCS)

4. GENITAL APPEARANCE AND EDUCATION: - Do you feel confident in assessing female anatomy genital appearance? o Not confident o Confident o Very confident

- How much teaching regarding female genital anatomy have you received in your medical training? - o none o a little o some o a lot

Please indicate what you think about Female Genital Cosmetic Surgery. (tick all that apply) o I have no opinion o If a woman wants this it is her choice o It should not be performed on women less than 18 years o A woman should be counselled first  o It is acceptable for cosmetic reasons 

o This surgery is unacceptable for cosmetic reasons  o It is no different to other types of cosmetic surgery

11. What is your opinion of the role of GPs for FGCS? ___________________________________________________________________________________ ___________________________________________________________________________________ ___________________________________________________________________________________ 12. Any final comments? ___________________________________________________________________________________
